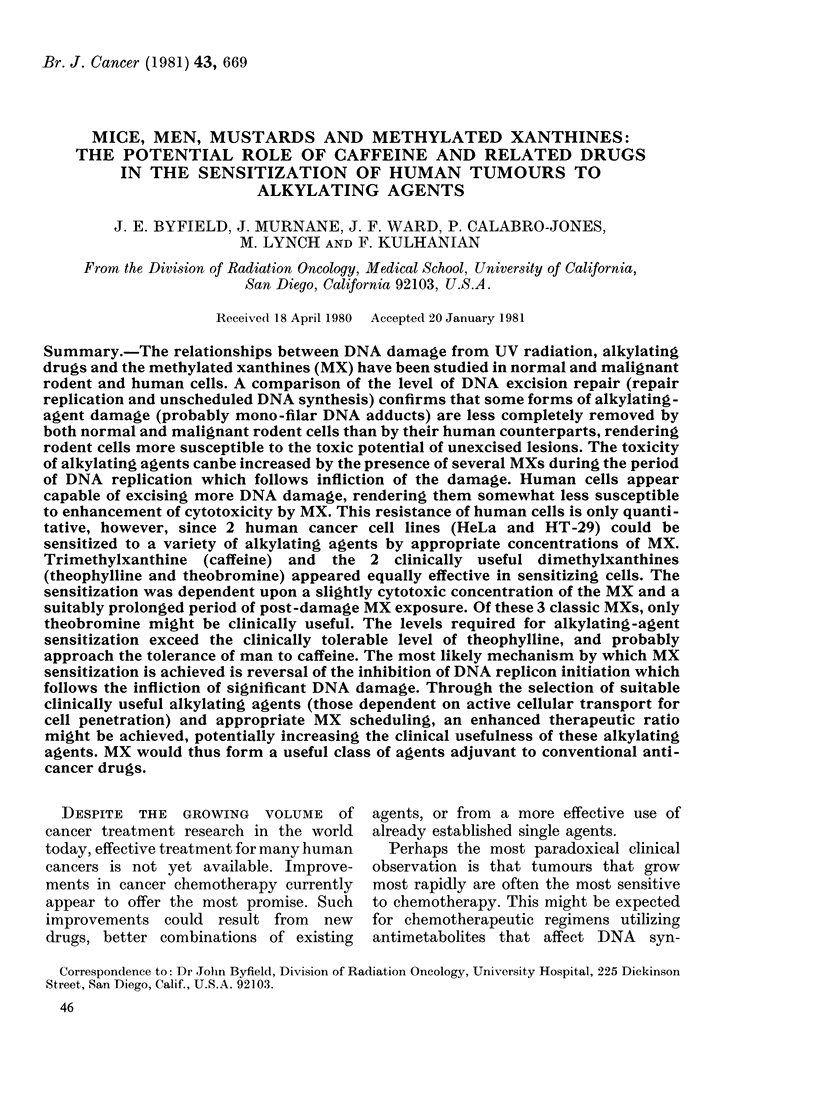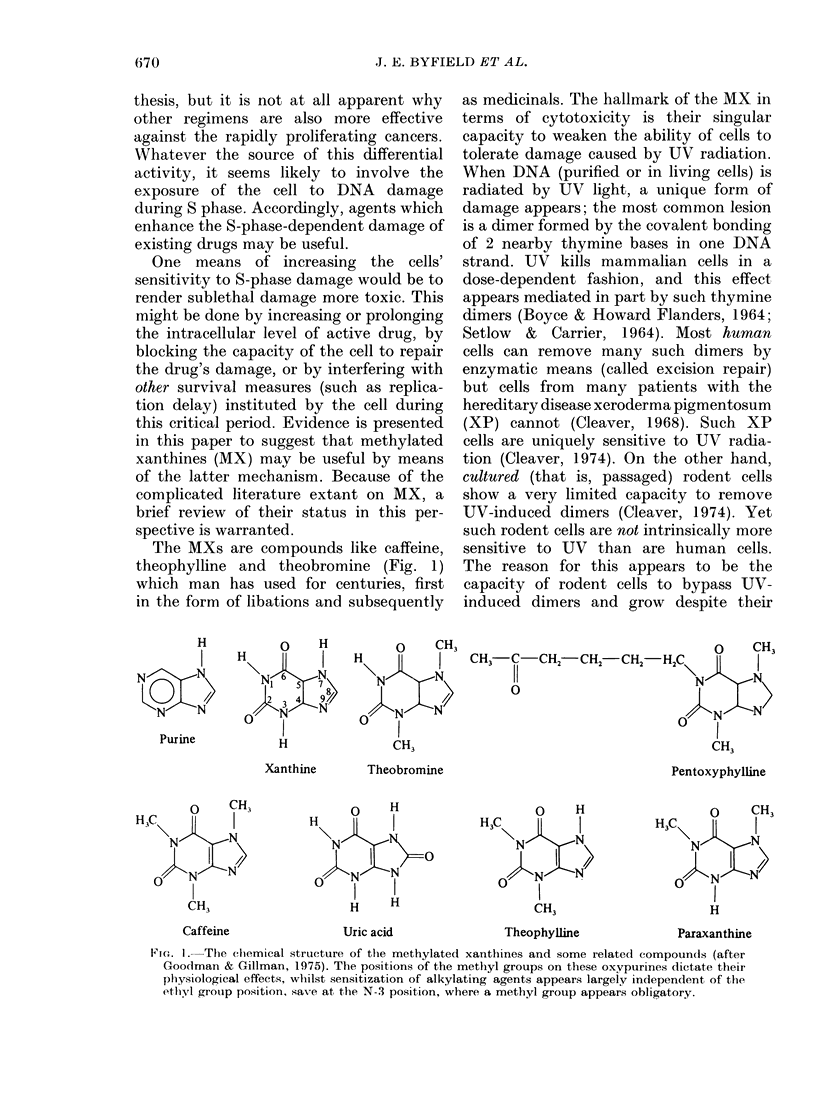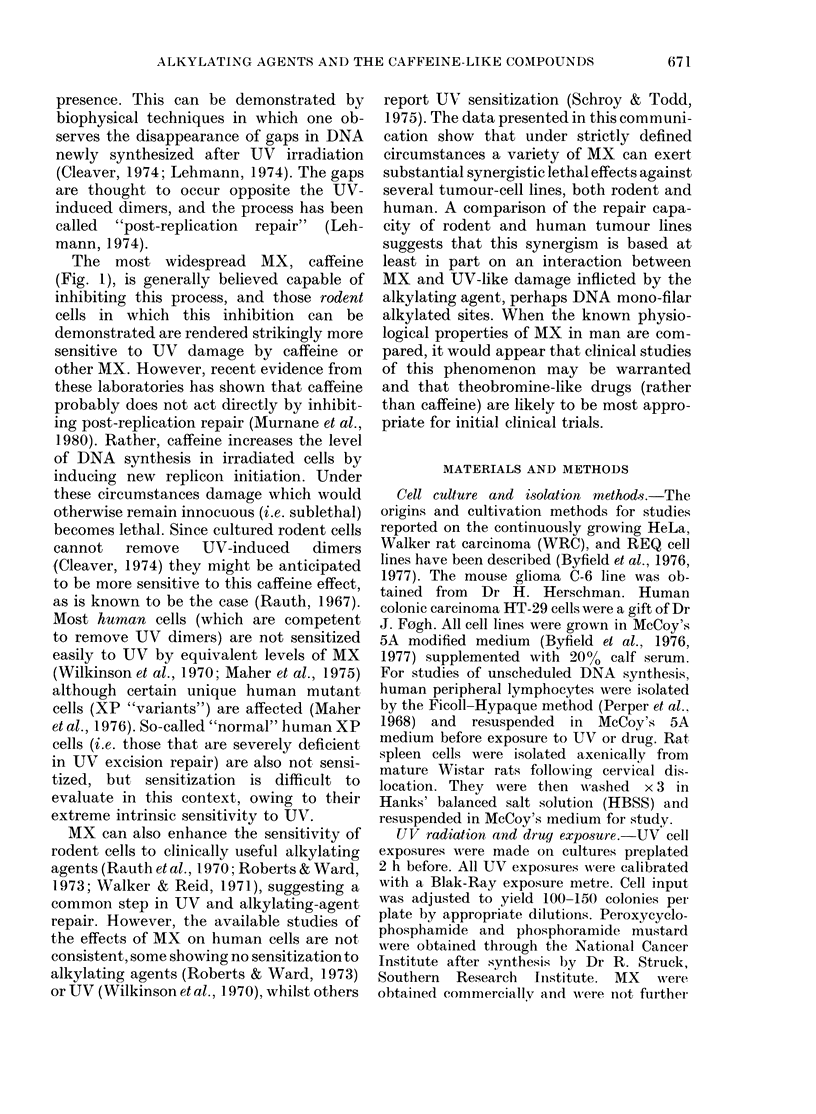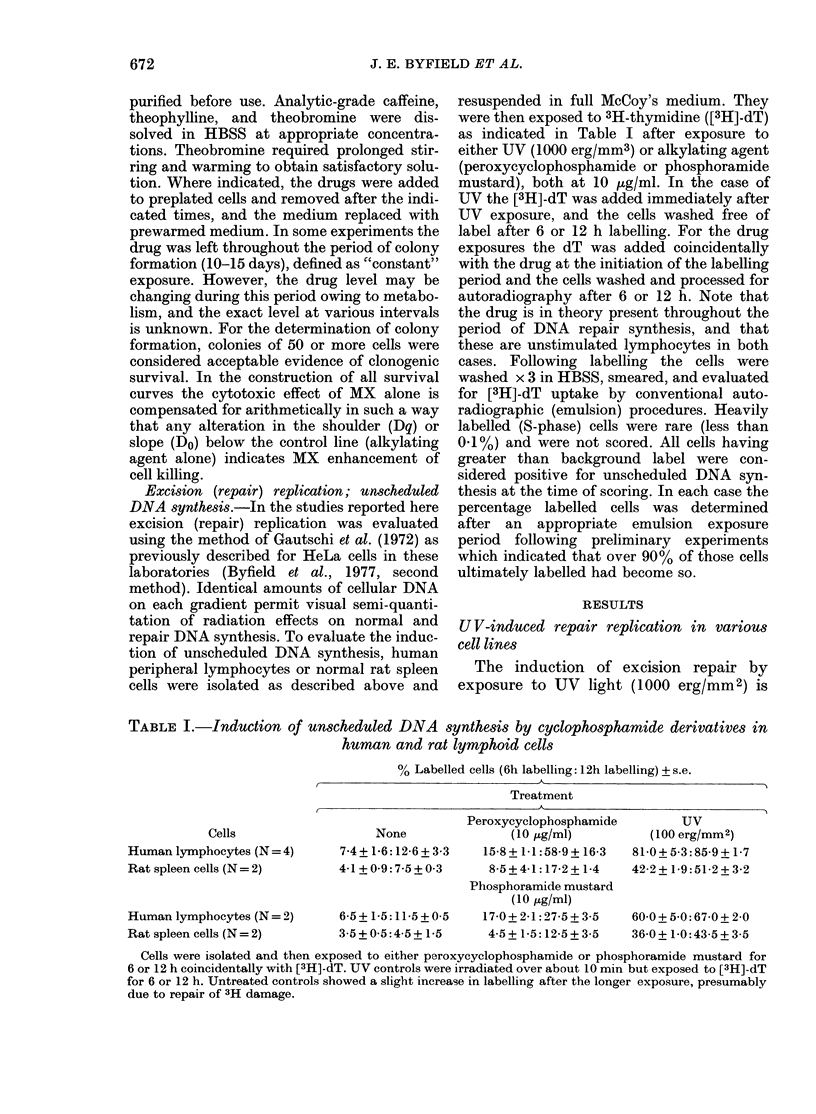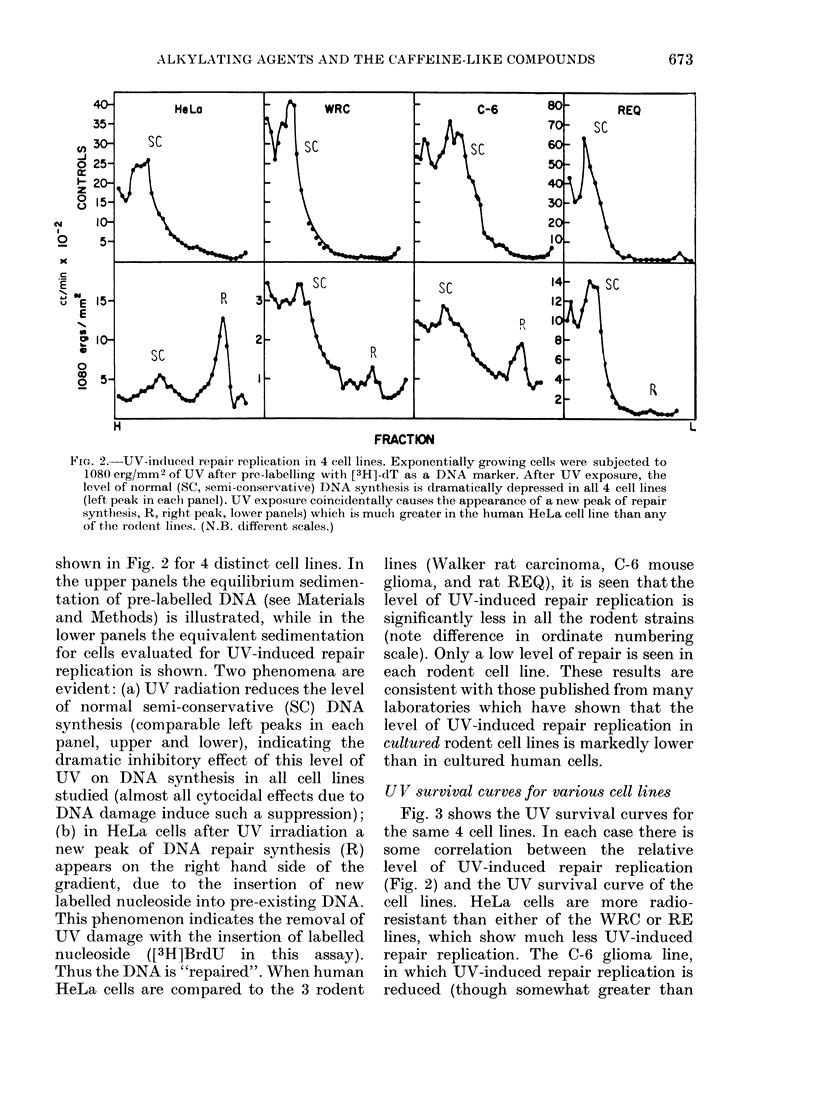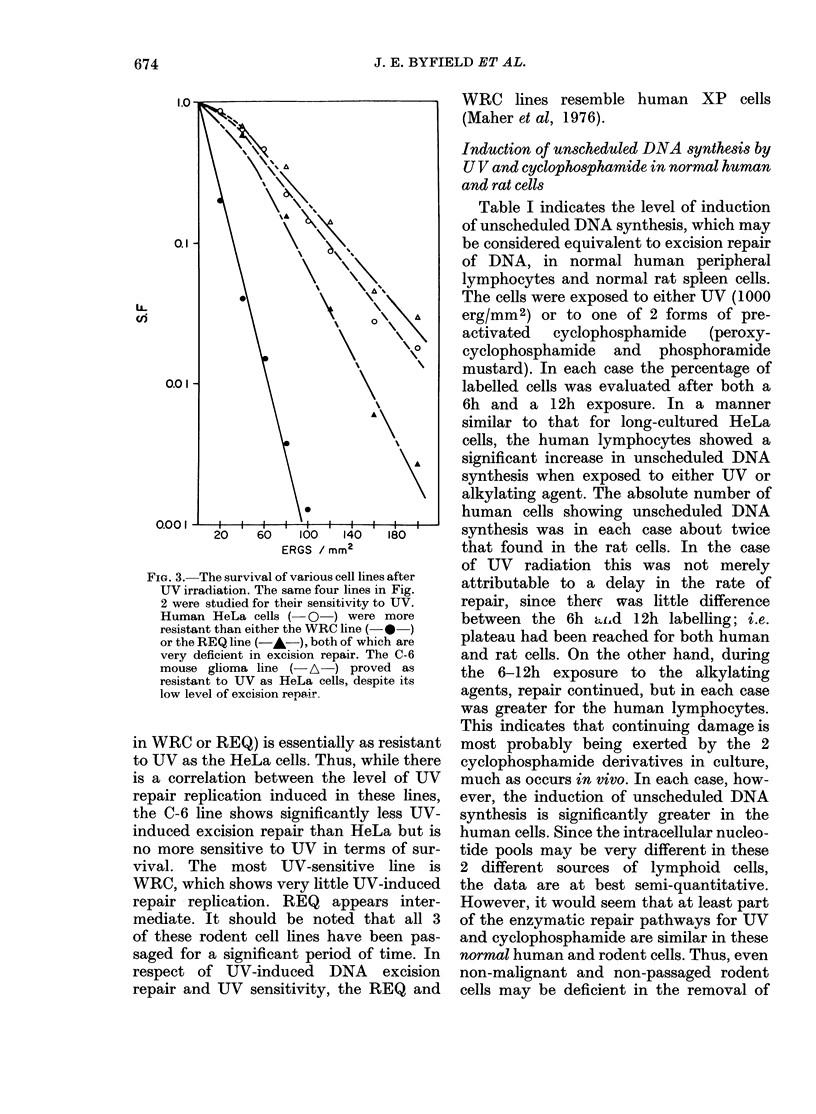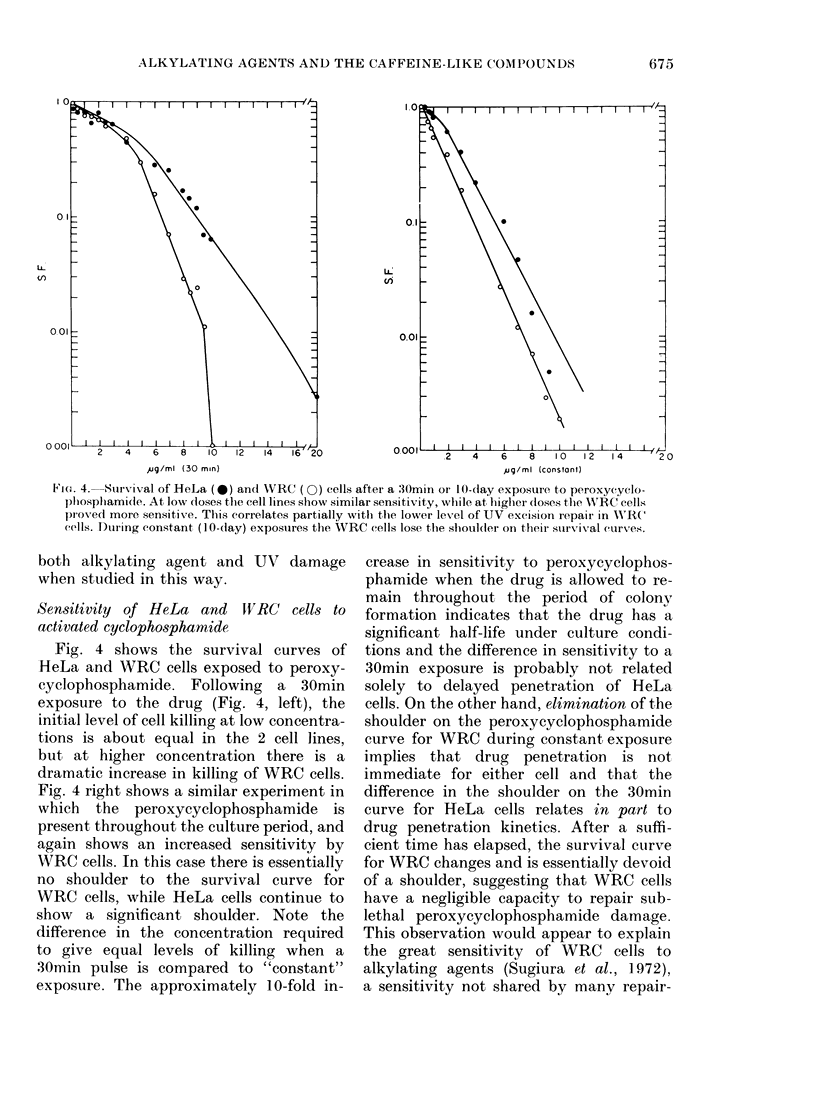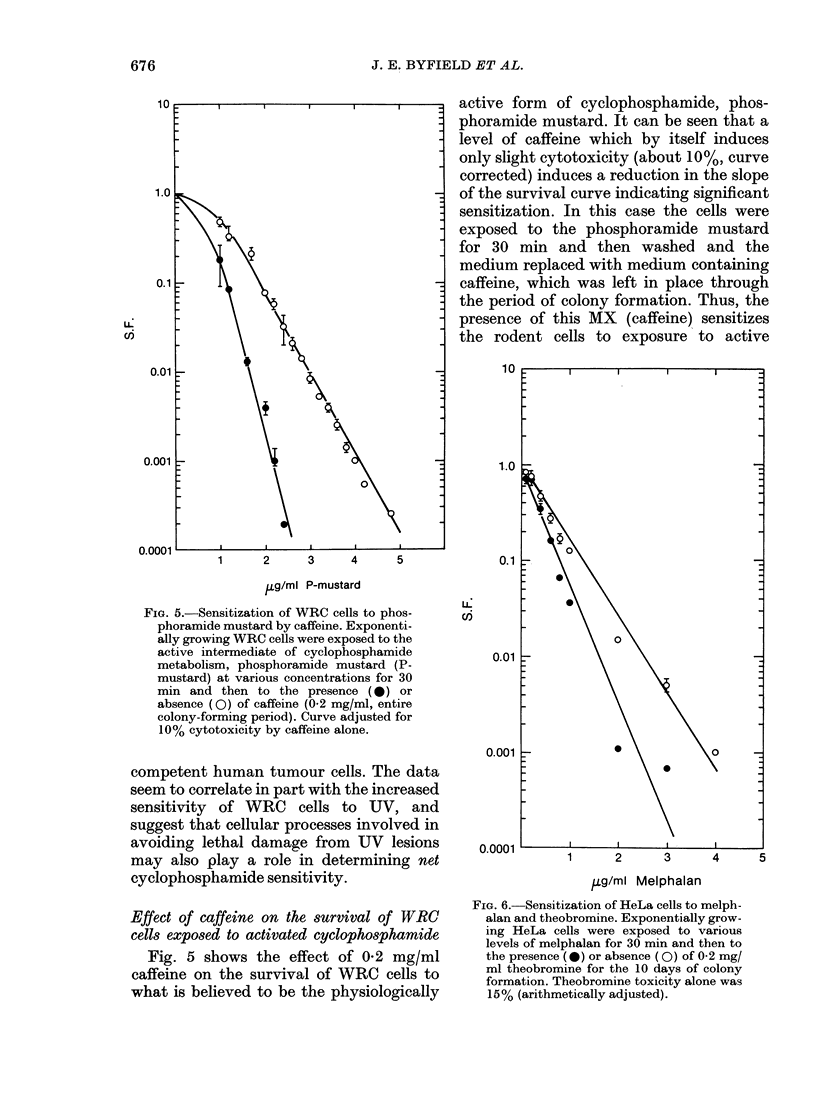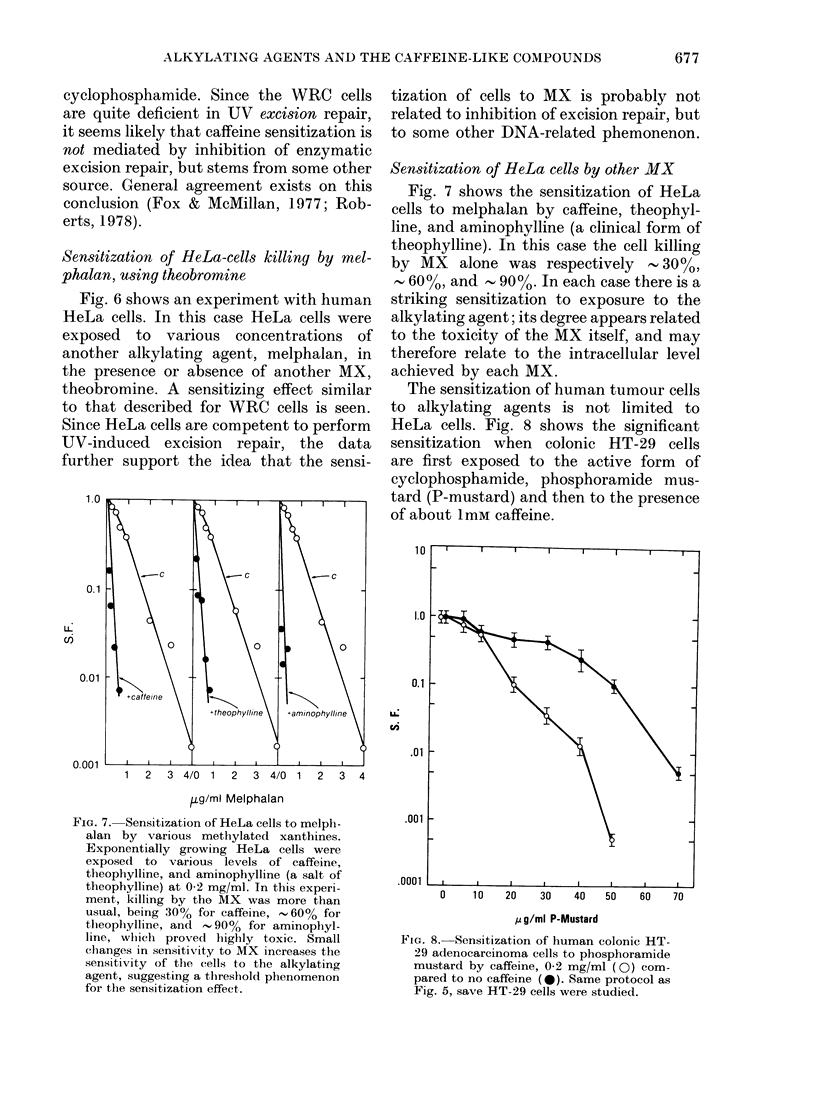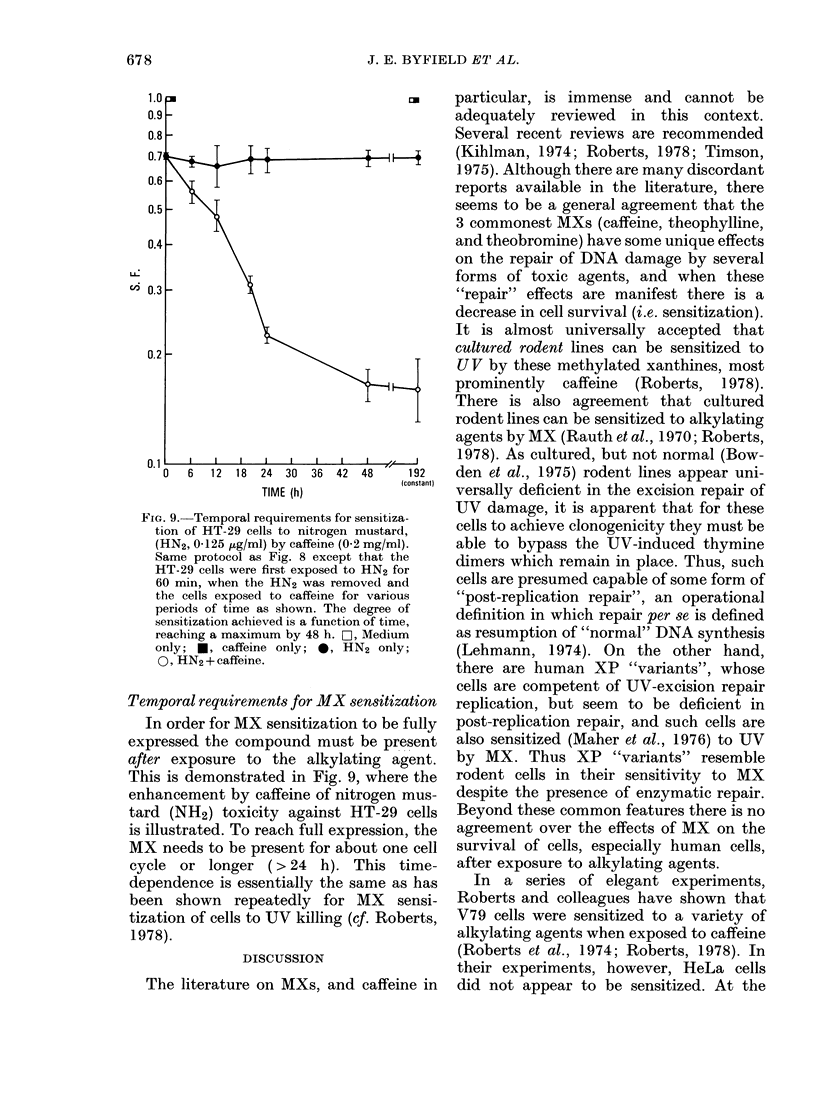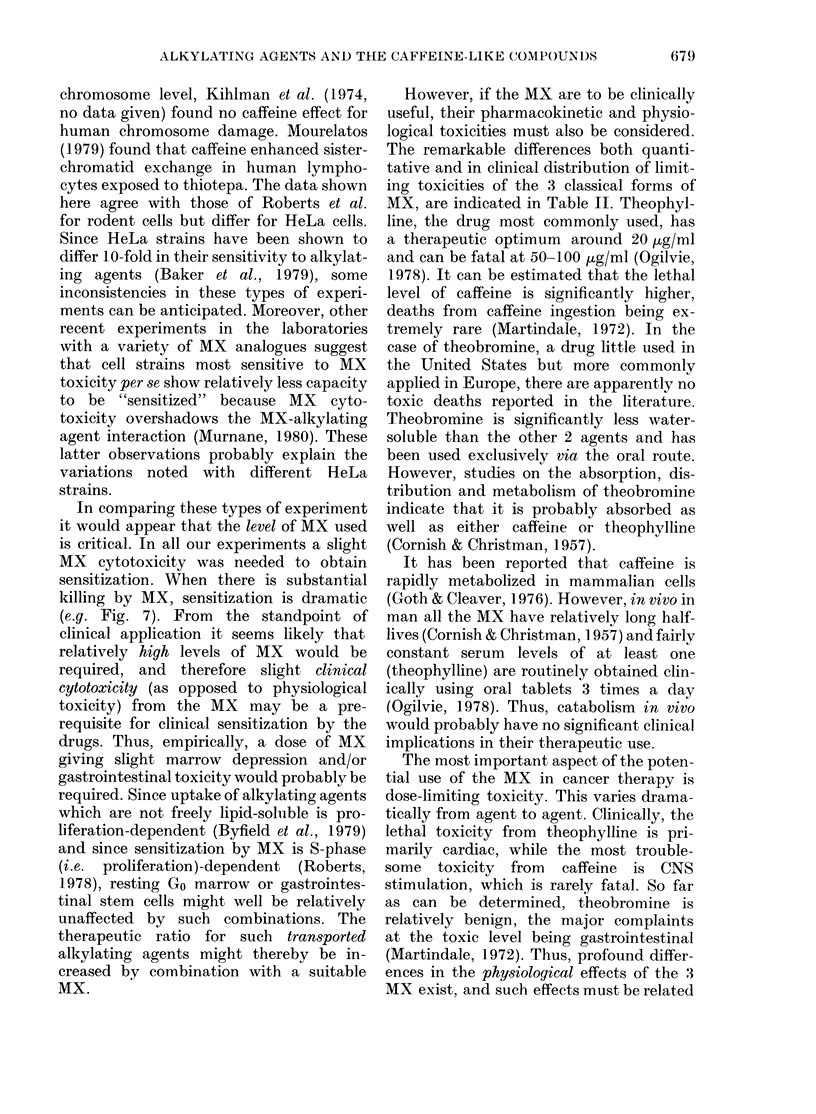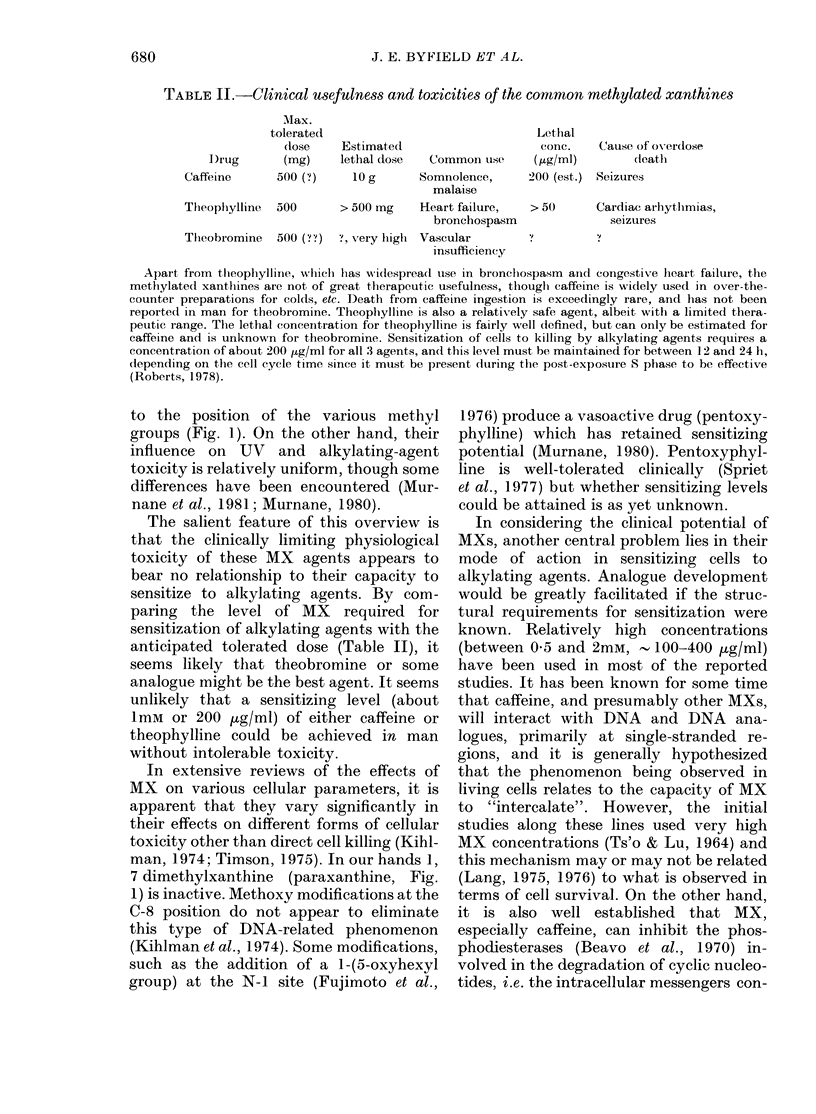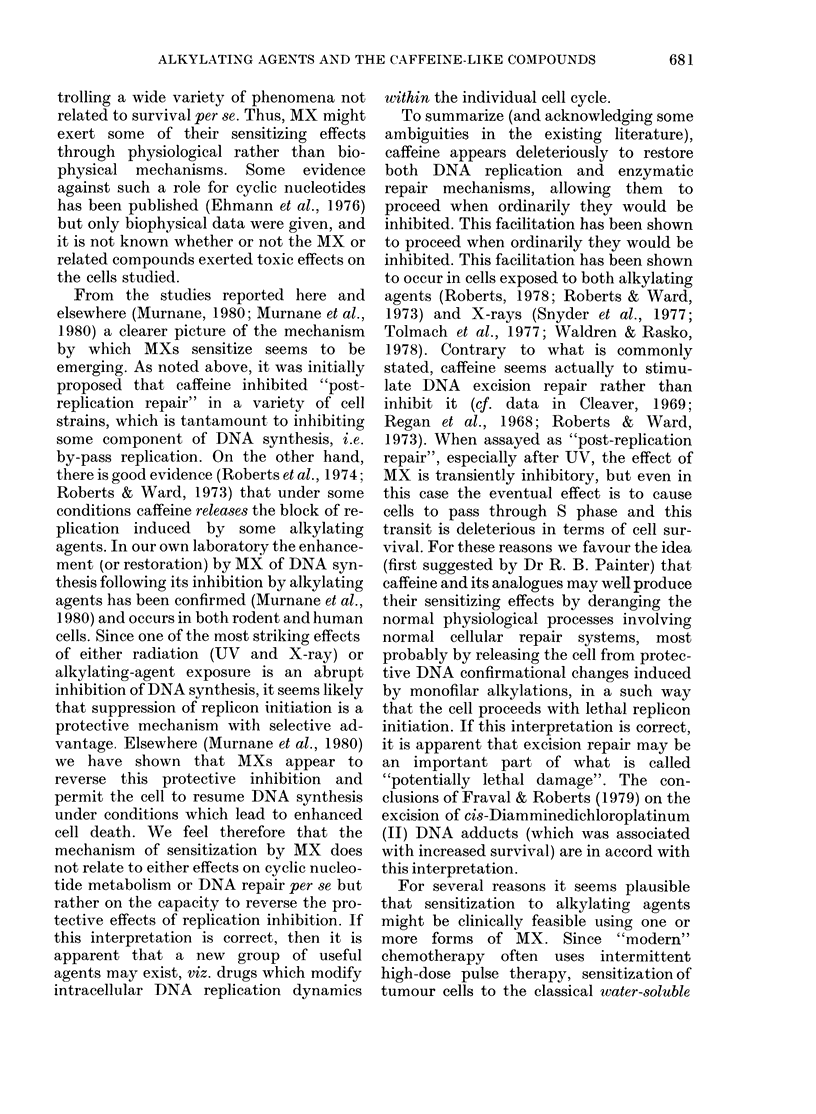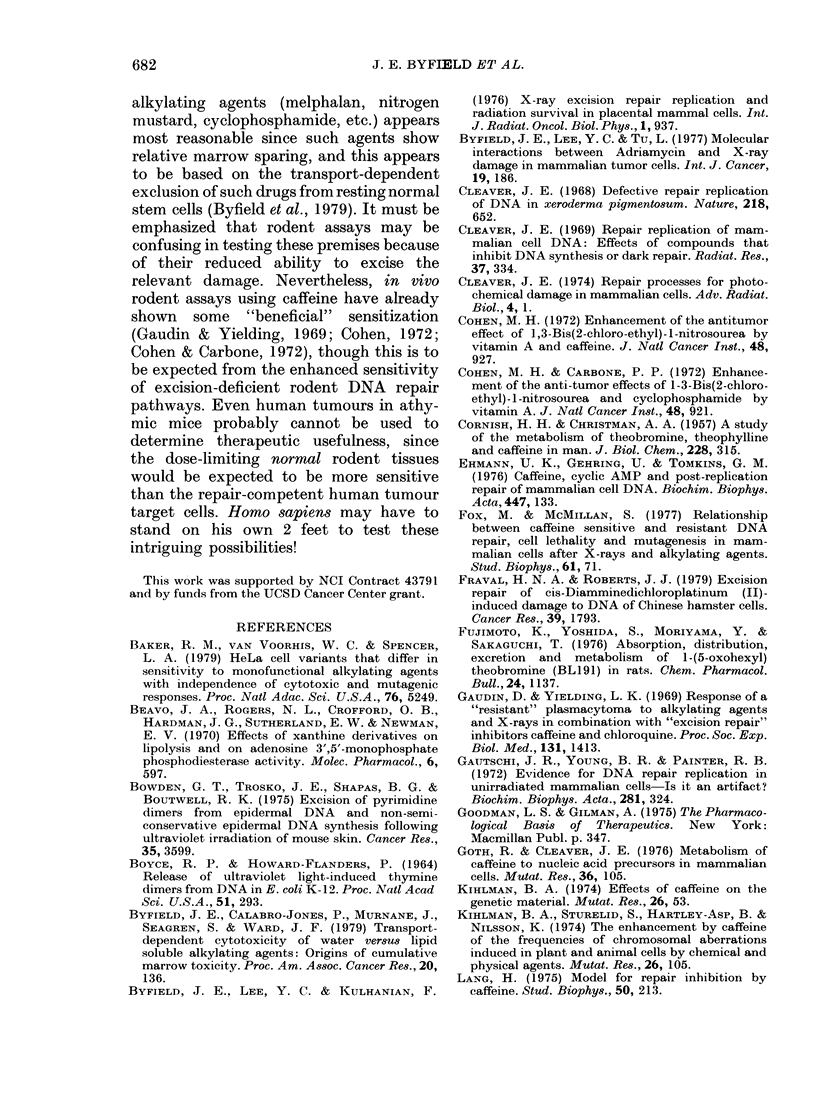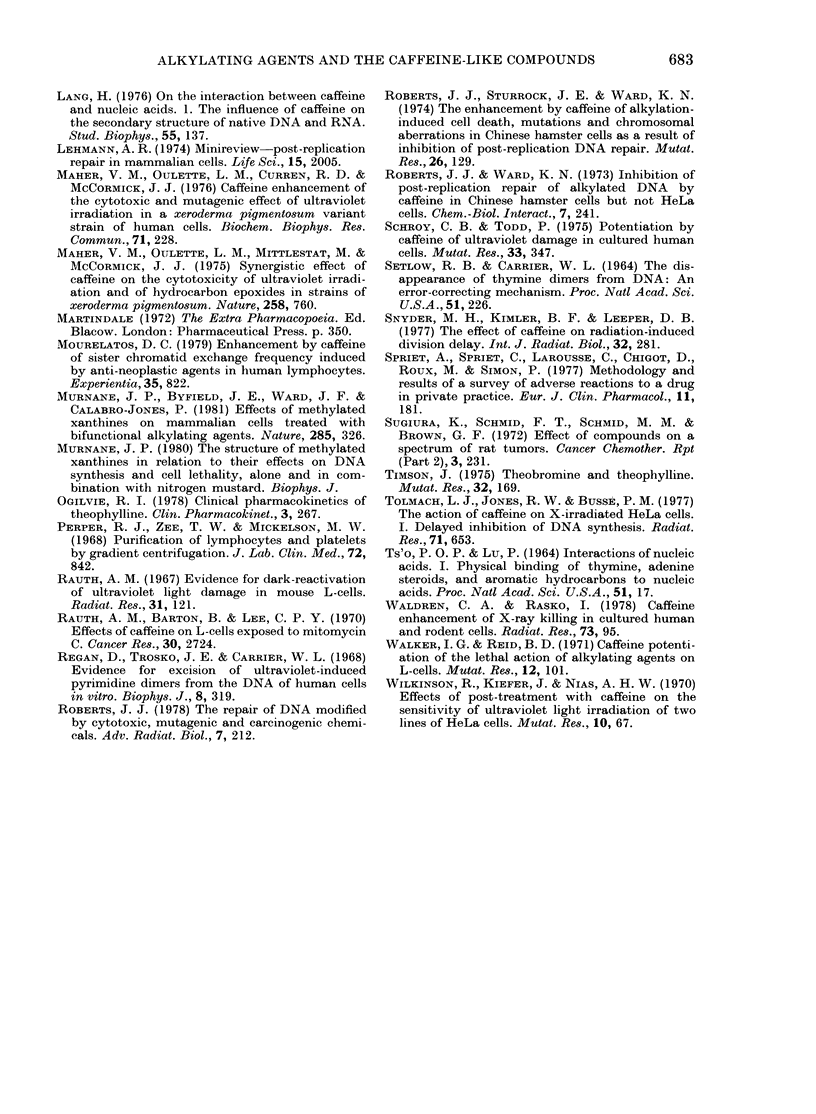# Mice, men, mustard and methylated xanthines: the potential role of caffeine and related drugs in the sensitization of human tumours to alkylating agents.

**DOI:** 10.1038/bjc.1981.98

**Published:** 1981-05

**Authors:** J. E. Byfield, J. Murnane, J. F. Ward, P. Calabro-Jones, M. Lynch, F. Kulhanian

## Abstract

The relationships between DNA damage from UV radiation, alkylating drugs and the methylated xanthines (MX) have been studied in normal and malignant rodent and human cells. A comparison of the level of DNA excision repair (repair replication and unscheduled DNA synthesis) confirms that some forms of alkylating-agent damage (probably mono-filar DNA adducts) are less completely removed by both normal and malignant rodent cells than by their human counterparts, rendering rodent cells more susceptible to the toxic potential of unexcised lesions. The toxicity of alkylating agents can be increased by the presence of several MXs during the period of DNA replication which follows infliction of the damage. Human cells appear capable of excising more DNA damage, rendering them somewhat less susceptible to enhancement of cytotoxicity by MX. This resistance of human cells is only quantitative, however, since 2 human cancer cell lines (HeLa and HT-29) could be sensitized to a variety of alkylating agents by appropriate concentrations of MX. Trimethylxanthine (caffeine) and the 2 clinically useful dimethylxanthines (theophylline and theobromine) appeared equally effective in sensitizing cells. The sensitization was dependent upon a slightly cytotoxic concentration of the MX and a suitably prolonged period of post-damage MX exposure. Of these 3 classic MXs, only theobromine might be clinically useful. The levels required for alkylating-agent sensitization exceed the clinically tolerable level of theophylline, and probably approach the tolerance of man to caffeine. The most likely mechanism by which MX sensitization is achieved is reversal of the inhibition of DNA replicon initiation which follows the infliction of significant DNA damage. Through the selection of suitable clinically useful alkylating agents (those dependent on active cellular transport for cell penetration) and appropriate MX scheduling, an enhanced therapeutic ratio might be achieved, potentially increasing the clinical usefulness of these alkylating agents. MX would thus form a useful class of agents adjuvant to conventional anti-cancer drugs.


Br. J. Cancer (1981) 43, 669

### MICE, MEN, MUSTARDS AND METHYLATED XANTHINES: THE POTENTIAL ROLE OF CAFFEINE AND RELATED DRUGS IN THE SENSITIZATION OF HUMAN TUMOURS TO ALKYLATING AGENTS

J. E. BYFIELD, J. MURNANE, J. F. WARD, P. CALABRO-JONES, M. LYNCH AND F. KULHANIAN

*From the Division of Radiation Oncology, Medical School, University of California, San Diego, California 92103, U.S.A.*

Received 18 April 1980 Accepted 20 January 1981

**Summary.—The relationships between DNA damage from UV radiation, alkylating drugs and the methylated xanthines (MX) have been studied in normal and malignant rodent and human cells. A comparison of the level of DNA excision repair (repair replication and unscheduled DNA synthesis) confirms that some forms of alkylating-agent damage (probably mono-filar DNA adducts) are less completely removed by both normal and malignant rodent cells than by their human counterparts, rendering rodent cells more susceptible to the toxic potential of unexcised lesions. The toxicity of alkylating agents canbe increased by the presence of several MXs during the period of DNA replication which follows infliction of the damage. Human cells appear capable of excising more DNA damage, rendering them somewhat less susceptible to enhancement of cytotoxicity by MX. This resistance of human cells is only quantitative, however, since 2 human cancer cell lines (HeLa and HT-29) could be sensitized to a variety of alkylating agents by appropriate concentrations of MX. Trimethylxanthine (caffeine) and the 2 clinically useful dimethylxanthines (theophylline and theobromine) appeared equally effective in sensitizing cells. The sensitization was dependent upon a slightly cytotoxic concentration of the MX and a suitably prolonged period of post-damage MX exposure. Of these 3 classic MXs, only theobromine might be clinically useful. The levels required for alkylating-agent sensitization exceed the clinically tolerable level of theophylline, and probably approach the tolerance of man to caffeine. The most likely mechanism by which MX sensitization is achieved is reversal of the inhibition of DNA replicon initiation which follows the infliction of significant DNA damage. Through the selection of suitable clinically useful alkylating agents (those dependent on active cellular transport for cell penetration) and appropriate MX scheduling, an enhanced therapeutic ratio might be achieved, potentially increasing the clinical usefulness of these alkylating agents. MX would thus form a useful class of agents adjuvant to conventional anti-cancer drugs.**

Despite the growing volume of cancer treatment research in the world today, effective treatment for many human cancers is not yet available. Improvements in cancer chemotherapy currently appear to offer the most promise. Such improvements could result from new drugs, better combinations of existing agents, or from a more effective use of already established single agents.

Perhaps the most paradoxical clinical observation is that tumours that grow most rapidly are often the most sensitive to chemotherapy. This might be expected for chemotherapeutic regimens utilizing antimetabolites that affect DNA syn-

Correspondence to: Dr John Byfield, Division of Radiation Oncology, University Hospital, 225 Dickinson Street, San Diego, Calif., U.S.A. 92103.

thesis, but it is not at all apparent why other regimens are also more effective against the rapidly proliferating cancers. Whatever the source of this differential activity, it seems likely to involve the exposure of the cell to DNA damage during S phase. Accordingly, agents which enhance the S-phase-dependent damage of existing drugs may be useful.

One means of increasing the cells' sensitivity to S-phase damage would be to render sublethal damage more toxic. This might be done by increasing or prolonging the intracellular level of active drug, by blocking the capacity of the cell to repair the drug's damage, or by interfering with *other* survival measures (such as replication delay) instituted by the cell during this critical period. Evidence is presented in this paper to suggest that methylated xanthines (MX) may be useful by means of the latter mechanism. Because of the complicated literature extant on MX, a brief review of their status in this perspective is warranted.

The MXs are compounds like caffeine, theophylline and theobromine (Fig. 1) which man has used for centuries, first in the form of libations and subsequently as medicinals. The hallmark of the MX in terms of cytotoxicity is their singular capacity to weaken the ability of cells to tolerate damage caused by UV radiation. When DNA (purified or in living cells) is radiated by UV light, a unique form of damage appears; the most common lesion is a dimer formed by the covalent bonding of 2 nearby thymine bases in one DNA strand. UV kills mammalian cells in a dose-dependent fashion, and this effect appears mediated in part by such thymine dimers (Boyce & Howard Flanders, 1964; Setlow & Carrier, 1964). Most *human* cells can remove many such dimers by enzymatic means (called excision repair) but cells from many patients with the hereditary disease xeroderma pigmentosum (XP) cannot (Cleaver, 1968). Such XP cells are uniquely sensitive to UV radiation (Cleaver, 1974). On the other hand, *cultured* (that is, passaged) rodent cells show a very limited capacity to remove UV-induced dimers (Cleaver, 1974). Yet such rodent cells are *not* intrinsically more sensitive to UV than are human cells. The reason for this appears to be the capacity of rodent cells to bypass UV-induced dimers and grow despite their

FIG. 1.—The chemical structure of the methylated xanthines and some related compounds (after Goodman & Gillman, 1975). The positions of the methyl groups on these oxypurines dictate their physiological effects, whilst sensitization of alkylating agents appears largely independent of the ethyl group position, save at the N-3 position, where a methyl group appears obligatory.

presence. This can be demonstrated by biophysical techniques in which one observes the disappearance of gaps in DNA newly synthesized after UV irradiation (Cleaver, 1974; Lehmann, 1974). The gaps are thought to occur opposite the UV-induced dimers, and the process has been called "post-replication repair" (Lehmann, 1974).

The most widespread MX, caffeine (Fig. 1), is generally believed capable of inhibiting this process, and those *rodent* cells in which this inhibition can be demonstrated are rendered strikingly more sensitive to UV damage by caffeine or other MX. However, recent evidence from these laboratories has shown that caffeine probably does not act directly by inhibiting post-replication repair (Murnane *et al.*, 1980). Rather, caffeine increases the level of DNA synthesis in irradiated cells by inducing new replicon initiation. Under these circumstances damage which would otherwise remain innocuous (*i.e.* sublethal) becomes lethal. Since cultured rodent cells cannot remove UV-induced dimers (Cleaver, 1974) they might be anticipated to be more sensitive to this caffeine effect, as is known to be the case (Rauth, 1967). Most *human* cells (which are competent to remove UV dimers) are not sensitized easily to UV by equivalent levels of MX (Wilkinson *et al.*, 1970; Maher *et al.*, 1975) although certain unique human mutant cells (XP "variants") are affected (Maher *et al.*, 1976). So-called "normal" human XP cells (*i.e.* those that are severely deficient in UV excision repair) are also not sensitized, but sensitization is difficult to evaluate in this context, owing to their extreme intrinsic sensitivity to UV.

MX can also enhance the sensitivity of rodent cells to clinically useful alkylating agents (Rauth *et al.*, 1970; Roberts & Ward, 1973; Walker & Reid, 1971), suggesting a common step in UV and alkylating-agent repair. However, the available studies of the effects of MX on human cells are not consistent, some showing no sensitization to alkylating agents (Roberts & Ward, 1973) or UV (Wilkinson *et al.*, 1970), whilst others report UV sensitization (Schroy & Todd, 1975). The data presented in this communication show that under strictly defined circumstances a variety of MX can exert substantial synergistic lethal effects against several tumour-cell lines, both rodent and human. A comparison of the repair capacity of rodent and human tumour lines suggests that this synergism is based at least in part on an interaction between MX and UV-like damage inflicted by the alkylating agent, perhaps DNA mono-filar alkylated sites. When the known physiological properties of MX in man are compared, it would appear that clinical studies of this phenomenon may be warranted and that theobromine-like drugs (rather than caffeine) are likely to be most appropriate for initial clinical trials.

### MATERIALS AND METHODS

*Cell culture and isolation methods*.—The origins and cultivation methods for studies reported on the continuously growing HeLa, Walker rat carcinoma (WRC), and REQ cell lines have been described (Byfield *et al.*, 1976, 1977). The mouse glioma C-6 line was obtained from Dr H. Herschman. Human colonic carcinoma HT-29 cells were a gift of Dr J. Føgh. All cell lines were grown in McCoy's 5A modified medium (Byfield *et al.*, 1976, 1977) supplemented with 20% calf serum. For studies of unscheduled DNA synthesis, human peripheral lymphocytes were isolated by the Ficoll–Hypaque method (Perper *et al.*, 1968) and resuspended in McCoy's 5A medium before exposure to UV or drug. Rat spleen cells were isolated axenically from mature Wistar rats following cervical dislocation. They were then washed ×3 in Hanks' balanced salt solution (HBSS) and resuspended in McCoy's medium for study.

*UV radiation and drug exposure*.—UV cell exposures were made on cultures preplated 2 h before. All UV exposures were calibrated with a Blak-Ray exposure metre. Cell input was adjusted to yield 100–150 colonies per plate by appropriate dilutions. Peroxycyclophosphamide and phosphoramide mustard were obtained through the National Cancer Institute after synthesis by Dr R. Struck, Southern Research Institute. MX were obtained commercially and were not further purified before use. Analytic-grade caffeine, theophylline, and theobromine were dissolved in HBSS at appropriate concentrations. Theobromine required prolonged stirring and warming to obtain satisfactory solution. Where indicated, the drugs were added to preplated cells and removed after the indicated times, and the medium replaced with prewarmed medium. In some experiments the drug was left throughout the period of colony formation (10–15 days), defined as "constant" exposure. However, the drug level may be changing during this period owing to metabolism, and the exact level at various intervals is unknown. For the determination of colony formation, colonies of 50 or more cells were considered acceptable evidence of clonogenic survival. In the construction of all survival curves the cytotoxic effect of MX alone is compensated for arithmetically in such a way that any alteration in the shoulder ($D_q$) or slope ($D_0$) below the control line (alkylating agent alone) indicates MX enhancement of cell killing.

*Excision (repair) replication; unscheduled DNA synthesis.*—In the studies reported here excision (repair) replication was evaluated using the method of Gautschi *et al.* (1972) as previously described for HeLa cells in these laboratories (Byfield *et al.*, 1977, second method). Identical amounts of cellular DNA on each gradient permit visual semi-quantitation of radiation effects on normal and repair DNA synthesis. To evaluate the induction of unscheduled DNA synthesis, human peripheral lymphocytes or normal rat spleen cells were isolated as described above and resuspended in full McCoy's medium. They were then exposed to $^3$H-thymidine ([$^3$H]-dT) as indicated in Table I after exposure to either UV (1000 erg/mm$^3$) or alkylating agent (peroxycyclophosphamide or phosphoramide mustard), both at 10 $\mu$g/ml. In the case of UV the [$^3$H]-dT was added immediately after UV exposure, and the cells washed free of label after 6 or 12 h labelling. For the drug exposures the dT was added coincidentally with the drug at the initiation of the labelling period and the cells washed and processed for autoradiography after 6 or 12 h. Note that the drug is in theory present throughout the period of DNA repair synthesis, and that these are unstimulated lymphocytes in both cases. Following labelling the cells were washed ×3 in HBSS, smeared, and evaluated for [$^3$H]-dT uptake by conventional autoradiographic (emulsion) procedures. Heavily labelled (S-phase) cells were rare (less than 0·1%) and were not scored. All cells having greater than background label were considered positive for unscheduled DNA synthesis at the time of scoring. In each case the percentage labelled cells was determined after an appropriate emulsion exposure period following preliminary experiments which indicated that over 90% of those cells ultimately labelled had become so.

### RESULTS

### *UV-induced repair replication in various cell lines*

The induction of excision repair by exposure to UV light (1000 erg/mm$^2$) is

TABLE I.—*Induction of unscheduled DNA synthesis by cyclophosphamide derivatives in human and rat lymphoid cells*

| | % Labelled cells (6h labelling: 12h labelling) ± s.e. | | |
|---|---|---|---|
| | Treatment | | |
| Cells | None | Peroxycyclophosphamide (10 μg/ml) | UV (100 erg/mm²) |
| Human lymphocytes (N = 4) | 7·4 ± 1·6 : 12·6 ± 3·3 | 15·8 ± 1·1 : 58·9 ± 16·3 | 81·0 ± 5·3 : 85·9 ± 1·7 |
| Rat spleen cells (N = 2) | 4·1 ± 0·9 : 7·5 ± 0·3 | 8·5 ± 4·1 : 17·2 ± 1·4 | 42·2 ± 1·9 : 51·2 ± 3·2 |
| | | Phosphoramide mustard (10 μg/ml) | |
| Human lymphocytes (N = 2) | 6·5 ± 1·5 : 11·5 ± 0·5 | 17·0 ± 2·1 : 27·5 ± 3·5 | 60·0 ± 5·0 : 67·0 ± 2·0 |
| Rat spleen cells (N = 2) | 3·5 ± 0·5 : 4·5 ± 1·5 | 4·5 ± 1·5 : 12·5 ± 3·5 | 36·0 ± 1·0 : 43·5 ± 3·5 |

Cells were isolated and then exposed to either peroxycyclophosphamide or phosphoramide mustard for 6 or 12 h coincidentally with [$^3$H]-dT. UV controls were irradiated over about 10 min but exposed to [$^3$H]-dT for 6 or 12 h. Untreated controls showed a slight increase in labelling after the longer exposure, presumably due to repair of $^3$H damage.

FIG. 2.—UV-induced repair replication in 4 cell lines. Exponentially growing cells were subjected to 1080 erg/mm$^2$ of UV after pre-labelling with [$^3$H]-dT as a DNA marker. After UV exposure, the level of normal (SC, semi-conservative) DNA synthesis is dramatically depressed in all 4 cell lines (left peak in each panel). UV exposure coincidentally causes the appearance of a new peak of repair synthesis, R, right peak, lower panels) which is much greater in the human HeLa cell line than any of the rodent lines. (N.B. different scales.)

shown in Fig. 2 for 4 distinct cell lines. In the upper panels the equilibrium sedimentation of pre-labelled DNA (see Materials and Methods) is illustrated, while in the lower panels the equivalent sedimentation for cells evaluated for UV-induced repair replication is shown. Two phenomena are evident: (a) UV radiation reduces the level of normal semi-conservative (SC) DNA synthesis (comparable left peaks in each panel, upper and lower), indicating the dramatic inhibitory effect of this level of UV on DNA synthesis in all cell lines studied (almost all cytocidal effects due to DNA damage induce such a suppression); (b) in HeLa cells after UV irradiation a new peak of DNA repair synthesis (R) appears on the right hand side of the gradient, due to the insertion of new labelled nucleoside into pre-existing DNA. This phenomenon indicates the removal of UV damage with the insertion of labelled nucleoside ([$^3$H]BrdU in this assay). Thus the DNA is "repaired". When human HeLa cells are compared to the 3 rodent lines (Walker rat carcinoma, C-6 mouse glioma, and rat REQ), it is seen that the level of UV-induced repair replication is significantly less in all the rodent strains (note difference in ordinate numbering scale). Only a low level of repair is seen in each rodent cell line. These results are consistent with those published from many laboratories which have shown that the level of UV-induced repair replication in *cultured* rodent cell lines is markedly lower than in cultured human cells.

*UV survival curves for various cell lines*

Fig. 3 shows the UV survival curves for the same 4 cell lines. In each case there is some correlation between the relative level of UV-induced repair replication (Fig. 2) and the UV survival curve of the cell lines. HeLa cells are more radio-resistant than either of the WRC or RE lines, which show much less UV-induced repair replication. The C-6 glioma line, in which UV-induced repair replication is reduced (though somewhat greater than

FIG. 3.—The survival of various cell lines after UV irradiation. The same four lines in Fig. 2 were studied for their sensitivity to UV. Human HeLa cells (—○—) were more resistant than either the WRC line (—●—) or the REQ line (—▲—), both of which are very deficient in excision repair. The C-6 mouse glioma line (—△—) proved as resistant to UV as HeLa cells, despite its low level of excision repair.

in WRC or REQ) is essentially as resistant to UV as the HeLa cells. Thus, while there is a correlation between the level of UV repair replication induced in these lines, the C-6 line shows significantly less UV-induced excision repair than HeLa but is no more sensitive to UV in terms of survival. The most UV-sensitive line is WRC, which shows very little UV-induced repair replication. REQ appears intermediate. It should be noted that all 3 of these rodent cell lines have been passaged for a significant period of time. In respect of UV-induced DNA excision repair and UV sensitivity, the REQ and WRC lines resemble human XP cells (Maher *et al*, 1976).

*Induction of unscheduled DNA synthesis by UV and cyclophosphamide in normal human and rat cells*

Table I indicates the level of induction of unscheduled DNA synthesis, which may be considered equivalent to excision repair of DNA, in normal human peripheral lymphocytes and normal rat spleen cells. The cells were exposed to either UV (1000 erg/mm$^2$) or to one of 2 forms of pre-activated cyclophosphamide (peroxy-cyclophosphamide and phosphoramide mustard). In each case the percentage of labelled cells was evaluated after both a 6h and a 12h exposure. In a manner similar to that for long-cultured HeLa cells, the human lymphocytes showed a significant increase in unscheduled DNA synthesis when exposed to either UV or alkylating agent. The absolute number of human cells showing unscheduled DNA synthesis was in each case about twice that found in the rat cells. In the case of UV radiation this was not merely attributable to a delay in the rate of repair, since there was little difference between the 6h and 12h labelling; *i.e.* plateau had been reached for both human and rat cells. On the other hand, during the 6–12h exposure to the alkylating agents, repair continued, but in each case was greater for the human lymphocytes. This indicates that continuing damage is most probably being exerted by the 2 cyclophosphamide derivatives in culture, much as occurs *in vivo*. In each case, however, the induction of unscheduled DNA synthesis is significantly greater in the human cells. Since the intracellular nucleotide pools may be very different in these 2 different sources of lymphoid cells, the data are at best semi-quantitative. However, it would seem that at least part of the enzymatic repair pathways for UV and cyclophosphamide are similar in these *normal* human and rodent cells. Thus, even non-malignant and non-passaged rodent cells may be deficient in the removal of

FIG. 4.—Survival of HeLa (●) and WRC (○) cells after a 30min or 10-day exposure to peroxycyclophosphamide. At low doses the cell lines show similar sensitivity, while at higher doses the WRC cells proved more sensitive. This correlates partially with the lower level of UV excision repair in WRC cells. During constant (10-day) exposures the WRC cells lose the shoulder on their survival curves.

both alkylating agent and UV damage when studied in this way.

*Sensitivity of HeLa and WRC cells to activated cyclophosphamide*

Fig. 4 shows the survival curves of HeLa and WRC cells exposed to peroxycyclophosphamide. Following a 30min exposure to the drug (Fig. 4, left), the initial level of cell killing at low concentrations is about equal in the 2 cell lines, but at higher concentration there is a dramatic increase in killing of WRC cells. Fig. 4 right shows a similar experiment in which the peroxycyclophosphamide is present throughout the culture period, and again shows an increased sensitivity by WRC cells. In this case there is essentially no shoulder to the survival curve for WRC cells, while HeLa cells continue to show a significant shoulder. Note the difference in the concentration required to give equal levels of killing when a 30min pulse is compared to "constant" exposure. The approximately 10-fold increase in sensitivity to peroxycyclophosphamide when the drug is allowed to remain throughout the period of colony formation indicates that the drug has a significant half-life under culture conditions and the difference in sensitivity to a 30min exposure is probably not related solely to delayed penetration of HeLa cells. On the other hand, *elimination* of the shoulder on the peroxycyclophosphamide curve for WRC during constant exposure implies that drug penetration is not immediate for either cell and that the difference in the shoulder on the 30min curve for HeLa cells relates *in part* to drug penetration kinetics. After a sufficient time has elapsed, the survival curve for WRC changes and is essentially devoid of a shoulder, suggesting that WRC cells have a negligible capacity to repair sublethal peroxycyclophosphamide damage. This observation would appear to explain the great sensitivity of WRC cells to alkylating agents (Sugiura *et al.*, 1972), a sensitivity not shared by many repair-

FIG. 5.—Sensitization of WRC cells to phosphoramide mustard by caffeine. Exponentially growing WRC cells were exposed to the active intermediate of cyclophosphamide metabolism, phosphoramide mustard (P-mustard) at various concentrations for 30 min and then to the presence (●) or absence (○) of caffeine (0·2 mg/ml, entire colony-forming period). Curve adjusted for 10% cytotoxicity by caffeine alone.

competent human tumour cells. The data seem to correlate in part with the increased sensitivity of WRC cells to UV, and suggest that cellular processes involved in avoiding lethal damage from UV lesions may also play a role in determining *net* cyclophosphamide sensitivity.

*Effect of caffeine on the survival of WRC cells exposed to activated cyclophosphamide*

Fig. 5 shows the effect of 0·2 mg/ml caffeine on the survival of WRC cells to what is believed to be the physiologically active form of cyclophosphamide, phosphoramide mustard. It can be seen that a level of caffeine which by itself induces only slight cytotoxicity (about 10%, curve corrected) induces a reduction in the slope of the survival curve indicating significant sensitization. In this case the cells were exposed to the phosphoramide mustard for 30 min and then washed and the medium replaced with medium containing caffeine, which was left in place through the period of colony formation. Thus, the presence of this MX (caffeine) sensitizes the rodent cells to exposure to active

FIG. 6.—Sensitization of HeLa cells to melphalan and theobromine. Exponentially growing HeLa cells were exposed to various levels of melphalan for 30 min and then to the presence (●) or absence (○) of 0·2 mg/ml theobromine for the 10 days of colony formation. Theobromine toxicity alone was 15% (arithmetically adjusted).

cyclophosphamide. Since the WRC cells are quite deficient in UV *excision* repair, it seems likely that caffeine sensitization is *not* mediated by inhibition of enzymatic excision repair, but stems from some other source. General agreement exists on this conclusion (Fox & McMillan, 1977; Roberts, 1978).

*Sensitization of HeLa-cells killing by melphalan, using theobromine*

Fig. 6 shows an experiment with human HeLa cells. In this case HeLa cells were exposed to various concentrations of another alkylating agent, melphalan, in the presence or absence of another MX, theobromine. A sensitizing effect similar to that described for WRC cells is seen. Since HeLa cells are competent to perform UV-induced excision repair, the data further support the idea that the sensitization of cells to MX is probably not related to inhibition of excision repair, but to some other DNA-related phemonenon.

*Sensitization of HeLa cells by other MX*

Fig. 7 shows the sensitization of HeLa cells to melphalan by caffeine, theophylline, and aminophylline (a clinical form of theophylline). In this case the cell killing by MX alone was respectively ~30%, ~60%, and ~90%. In each case there is a striking sensitization to exposure to the alkylating agent; its degree appears related to the toxicity of the MX itself, and may therefore relate to the intracellular level achieved by each MX.

The sensitization of human tumour cells to alkylating agents is not limited to HeLa cells. Fig. 8 shows the significant sensitization when colonic HT-29 cells are first exposed to the active form of cyclophosphamide, phosphoramide mustard (P-mustard) and then to the presence of about 1mM caffeine.

FIG. 7.—Sensitization of HeLa cells to melphalan by various methylated xanthines. Exponentially growing HeLa cells were exposed to various levels of caffeine, theophylline, and aminophylline (a salt of theophylline) at 0·2 mg/ml. In this experiment, killing by the MX was more than usual, being 30% for caffeine, ~60% for theophylline, and ~90% for aminophylline, which proved highly toxic. Small changes in sensitivity to MX increases the sensitivity of the cells to the alkylating agent, suggesting a threshold phenomenon for the sensitization effect.

FIG. 8.—Sensitization of human colonic HT-29 adenocarcinoma cells to phosphoramide mustard by caffeine, 0·2 mg/ml (○) compared to no caffeine (●). Same protocol as Fig. 5, save HT-29 cells were studied.

FIG. 9.—Temporal requirements for sensitization of HT-29 cells to nitrogen mustard, ($HN_2$, 0·125 μg/ml) by caffeine (0·2 mg/ml). Same protocol as Fig. 8 except that the HT-29 cells were first exposed to $HN_2$ for 60 min, when the $HN_2$ was removed and the cells exposed to caffeine for various periods of time as shown. The degree of sensitization achieved is a function of time, reaching a maximum by 48 h. □, Medium only; ■, caffeine only; ●, $HN_2$ only; ○, $HN_2$+caffeine.

*Temporal requirements for MX sensitization*

In order for MX sensitization to be fully expressed the compound must be present *after* exposure to the alkylating agent. This is demonstrated in Fig. 9, where the enhancement by caffeine of nitrogen mustard ($NH_2$) toxicity against HT-29 cells is illustrated. To reach full expression, the MX needs to be present for about one cell cycle or longer (>24 h). This time-dependence is essentially the same as has been shown repeatedly for MX sensitization of cells to UV killing (*cf.* Roberts, 1978).

DISCUSSION

The literature on MXs, and caffeine in particular, is immense and cannot be adequately reviewed in this context. Several recent reviews are recommended (Kihlman, 1974; Roberts, 1978; Timson, 1975). Although there are many discordant reports available in the literature, there seems to be a general agreement that the 3 commonest MXs (caffeine, theophylline, and theobromine) have some unique effects on the repair of DNA damage by several forms of toxic agents, and when these "repair" effects are manifest there is a decrease in cell survival (*i.e.* sensitization). It is almost universally accepted that *cultured rodent* lines can be sensitized to *UV* by these methylated xanthines, most prominently caffeine (Roberts, 1978). There is also agreement that cultured rodent lines can be sensitized to alkylating agents by MX (Rauth *et al.*, 1970; Roberts, 1978). As cultured, but not normal (Bowden *et al.*, 1975) rodent lines appear universally deficient in the excision repair of UV damage, it is apparent that for these cells to achieve clonogenicity they must be able to bypass the UV-induced thymine dimers which remain in place. Thus, such cells are presumed capable of some form of "post-replication repair", an operational definition in which repair *per se* is defined as resumption of "normal" DNA synthesis (Lehmann, 1974). On the other hand, there are human XP "variants", whose cells are competent of UV-excision repair replication, but seem to be deficient in post-replication repair, and such cells are also sensitized (Maher *et al.*, 1976) to UV by MX. Thus XP "variants" resemble rodent cells in their sensitivity to MX despite the presence of enzymatic repair. Beyond these common features there is no agreement over the effects of MX on the survival of cells, especially human cells, after exposure to alkylating agents.

In a series of elegant experiments, Roberts and colleagues have shown that V79 cells were sensitized to a variety of alkylating agents when exposed to caffeine (Roberts *et al.*, 1974; Roberts, 1978). In their experiments, however, HeLa cells did not appear to be sensitized. At the chromosome level, Kihlman *et al.* (1974, no data given) found no caffeine effect for human chromosome damage. Mourelatos (1979) found that caffeine enhanced sister-chromatid exchange in human lymphocytes exposed to thiotepa. The data shown here agree with those of Roberts *et al.* for rodent cells but differ for HeLa cells. Since HeLa strains have been shown to differ 10-fold in their sensitivity to alkylating agents (Baker *et al.*, 1979), some inconsistencies in these types of experiments can be anticipated. Moreover, other recent experiments in the laboratories with a variety of MX analogues suggest that cell strains most sensitive to MX toxicity *per se* show relatively less capacity to be "sensitized" because MX cytotoxicity overshadows the MX-alkylating agent interaction (Murnane, 1980). These latter observations probably explain the variations noted with different HeLa strains.

In comparing these types of experiment it would appear that the *level* of MX used is critical. In all our experiments a slight MX cytotoxicity was needed to obtain sensitization. When there is substantial killing by MX, sensitization is dramatic (*e.g.* Fig. 7). From the standpoint of clinical application it seems likely that relatively *high* levels of MX would be required, and therefore slight *clinical cytotoxicity* (as opposed to physiological toxicity) from the MX may be a prerequisite for clinical sensitization by the drugs. Thus, empirically, a dose of MX giving slight marrow depression and/or gastrointestinal toxicity would probably be required. Since uptake of alkylating agents which are not freely lipid-soluble is proliferation-dependent (Byfield *et al.*, 1979) and since sensitization by MX is S-phase (*i.e.* proliferation)-dependent (Roberts, 1978), resting $G_0$ marrow or gastrointestinal stem cells might well be relatively unaffected by such combinations. The therapeutic ratio for such *transported* alkylating agents might thereby be increased by combination with a suitable MX.

However, if the MX are to be clinically useful, their pharmacokinetic and physiological toxicities must also be considered. The remarkable differences both quantitative and in clinical distribution of limiting toxicities of the 3 classical forms of MX, are indicated in Table II. Theophylline, the drug most commonly used, has a therapeutic optimum around 20 $\mu$g/ml and can be fatal at 50–100 $\mu$g/ml (Ogilvie, 1978). It can be estimated that the lethal level of caffeine is significantly higher, deaths from caffeine ingestion being extremely rare (Martindale, 1972). In the case of theobromine, a drug little used in the United States but more commonly applied in Europe, there are apparently no toxic deaths reported in the literature. Theobromine is significantly less water-soluble than the other 2 agents and has been used exclusively *via* the oral route. However, studies on the absorption, distribution and metabolism of theobromine indicate that it is probably absorbed as well as either caffeine or theophylline (Cornish & Christman, 1957).

It has been reported that caffeine is rapidly metabolized in mammalian cells (Goth & Cleaver, 1976). However, *in vivo* in man all the MX have relatively long half-lives (Cornish & Christman, 1957) and fairly constant serum levels of at least one (theophylline) are routinely obtained clinically using oral tablets 3 times a day (Ogilvie, 1978). Thus, catabolism *in vivo* would probably have no significant clinical implications in their therapeutic use.

The most important aspect of the potential use of the MX in cancer therapy is dose-limiting toxicity. This varies dramatically from agent to agent. Clinically, the lethal toxicity from theophylline is primarily cardiac, while the most troublesome toxicity from caffeine is CNS stimulation, which is rarely fatal. So far as can be determined, theobromine is relatively benign, the major complaints at the toxic level being gastrointestinal (Martindale, 1972). Thus, profound differences in the *physiological* effects of the 3 MX exist, and such effects must be related

TABLE II.—*Clinical usefulness and toxicities of the common methylated xanthines*

| Drug | Max. tolerated dose (mg) | Estimated lethal dose | Common use | Lethal conc. ($\mu$g/ml) | Cause of overdose death |
|---|---|---|---|---|---|
| Caffeine | 500 (?) | 10 g | Somnolence, malaise | 200 (est.) | Seizures |
| Theophylline | 500 | > 500 mg | Heart failure, bronchospasm | > 50 | Cardiac arhythmias, seizures |
| Theobromine | 500 (??) | ?, very high | Vascular insufficiency | ? | ? |

Apart from theophylline, which has widespread use in bronchospasm and congestive heart failure, the methylated xanthines are not of great therapeutic usefulness, though caffeine is widely used in over-the-counter preparations for colds, *etc.* Death from caffeine ingestion is exceedingly rare, and has not been reported in man for theobromine. Theophylline is also a relatively safe agent, albeit with a limited therapeutic range. The lethal concentration for theophylline is fairly well defined, but can only be estimated for caffeine and is unknown for theobromine. Sensitization of cells to killing by alkylating agents requires a concentration of about 200 $\mu$g/ml for all 3 agents, and this level must be maintained for between 12 and 24 h, depending on the cell cycle time since it must be present during the post-exposure S phase to be effective (Roberts, 1978).

to the position of the various methyl groups (Fig. 1). On the other hand, their influence on UV and alkylating-agent toxicity is relatively uniform, though some differences have been encountered (Murnane *et al.*, 1981; Murnane, 1980).

The salient feature of this overview is that the clinically limiting physiological toxicity of these MX agents appears to bear no relationship to their capacity to sensitize to alkylating agents. By comparing the level of MX required for sensitization of alkylating agents with the anticipated tolerated dose (Table II), it seems likely that theobromine or some analogue might be the best agent. It seems unlikely that a sensitizing level (about 1mM or 200 $\mu$g/ml) of either caffeine or theophylline could be achieved *in* man without intolerable toxicity.

In extensive reviews of the effects of MX on various cellular parameters, it is apparent that they vary significantly in their effects on different forms of cellular toxicity other than direct cell killing (Kihlman, 1974; Timson, 1975). In our hands 1, 7 dimethylxanthine (paraxanthine, Fig. 1) is inactive. Methoxy modifications at the C-8 position do not appear to eliminate this type of DNA-related phenomenon (Kihlman *et al.*, 1974). Some modifications, such as the addition of a 1-(5-oxyhexyl group) at the N-1 site (Fujimoto *et al.*, 1976) produce a vasoactive drug (pentoxyphylline) which has retained sensitizing potential (Murnane, 1980). Pentoxyphylline is well-tolerated clinically (Spriet *et al.*, 1977) but whether sensitizing levels could be attained is as yet unknown.

In considering the clinical potential of MXs, another central problem lies in their mode of action in sensitizing cells to alkylating agents. Analogue development would be greatly facilitated if the structural requirements for sensitization were known. Relatively high concentrations (between 0·5 and 2mM, ~100–400 $\mu$g/ml) have been used in most of the reported studies. It has been known for some time that caffeine, and presumably other MXs, will interact with DNA and DNA analogues, primarily at single-stranded regions, and it is generally hypothesized that the phenomenon being observed in living cells relates to the capacity of MX to "intercalate". However, the initial studies along these lines used very high MX concentrations (Ts'o & Lu, 1964) and this mechanism may or may not be related (Lang, 1975, 1976) to what is observed in terms of cell survival. On the other hand, it is also well established that MX, especially caffeine, can inhibit the phosphodiesterases (Beavo *et al.*, 1970) involved in the degradation of cyclic nucleotides, *i.e.* the intracellular messengers controlling a wide variety of phenomena not related to survival *per se*. Thus, MX might exert some of their sensitizing effects through physiological rather than biophysical mechanisms. Some evidence against such a role for cyclic nucleotides has been published (Ehmann *et al.*, 1976) but only biophysical data were given, and it is not known whether or not the MX or related compounds exerted toxic effects on the cells studied.

From the studies reported here and elsewhere (Murnane, 1980; Murnane *et al.*, 1980) a clearer picture of the mechanism by which MXs sensitize seems to be emerging. As noted above, it was initially proposed that caffeine inhibited "post-replication repair" in a variety of cell strains, which is tantamount to inhibiting some component of DNA synthesis, *i.e.* by-pass replication. On the other hand, there is good evidence (Roberts *et al.*, 1974; Roberts & Ward, 1973) that under some conditions caffeine *releases* the block of replication induced by some alkylating agents. In our own laboratory the enhancement (or restoration) by MX of DNA synthesis following its inhibition by alkylating agents has been confirmed (Murnane *et al.*, 1980) and occurs in both rodent and human cells. Since one of the most striking effects of either radiation (UV and X-ray) or alkylating-agent exposure is an abrupt inhibition of DNA synthesis, it seems likely that suppression of replicon initiation is a protective mechanism with selective advantage. Elsewhere (Murnane *et al.*, 1980) we have shown that MXs appear to reverse this protective inhibition and permit the cell to resume DNA synthesis under conditions which lead to enhanced cell death. We feel therefore that the mechanism of sensitization by MX does not relate to either effects on cyclic nucleotide metabolism or DNA repair *per se* but rather on the capacity to reverse the protective effects of replication inhibition. If this interpretation is correct, then it is apparent that a new group of useful agents may exist, *viz.* drugs which modify intracellular DNA replication dynamics *within* the individual cell cycle.

To summarize (and acknowledging some ambiguities in the existing literature), caffeine appears deleteriously to restore both DNA replication and enzymatic repair mechanisms, allowing them to proceed when ordinarily they would be inhibited. This facilitation has been shown to proceed when ordinarily they would be inhibited. This facilitation has been shown to occur in cells exposed to both alkylating agents (Roberts, 1978; Roberts & Ward, 1973) and X-rays (Snyder *et al.*, 1977; Tolmach *et al.*, 1977; Waldren & Rasko, 1978). Contrary to what is commonly stated, caffeine seems actually to stimulate DNA excision repair rather than inhibit it (*cf.* data in Cleaver, 1969; Regan *et al.*, 1968; Roberts & Ward, 1973). When assayed as "post-replication repair", especially after UV, the effect of MX is transiently inhibitory, but even in this case the eventual effect is to cause cells to pass through S phase and this transit is deleterious in terms of cell survival. For these reasons we favour the idea (first suggested by Dr R. B. Painter) that caffeine and its analogues may well produce their sensitizing effects by deranging the normal physiological processes involving normal cellular repair systems, most probably by releasing the cell from protective DNA confirmational changes induced by monofilar alkylations, in a such way that the cell proceeds with lethal replicon initiation. If this interpretation is correct, it is apparent that excision repair may be an important part of what is called "potentially lethal damage". The conclusions of Fraval & Roberts (1979) on the excision of *cis*-Diamminedichloroplatinum (II) DNA adducts (which was associated with increased survival) are in accord with this interpretation.

For several reasons it seems plausible that sensitization to alkylating agents might be clinically feasible using one or more forms of MX. Since "modern" chemotherapy often uses intermittent high-dose pulse therapy, sensitization of tumour cells to the classical *water-soluble*

alkylating agents (melphalan, nitrogen mustard, cyclophosphamide, etc.) appears most reasonable since such agents show relative marrow sparing, and this appears to be based on the transport-dependent exclusion of such drugs from resting normal stem cells (Byfield *et al.*, 1979). It must be emphasized that rodent assays may be confusing in testing these premises because of their reduced ability to excise the relevant damage. Nevertheless, *in vivo* rodent assays using caffeine have already shown some "beneficial" sensitization (Gaudin & Yielding, 1969; Cohen, 1972; Cohen & Carbone, 1972), though this is to be expected from the enhanced sensitivity of excision-deficient rodent DNA repair pathways. Even human tumours in athymic mice probably cannot be used to determine therapeutic usefulness, since the dose-limiting *normal* rodent tissues would be expected to be more sensitive than the repair-competent human tumour target cells. *Homo sapiens* may have to stand on his own 2 feet to test these intriguing possibilities!

This work was supported by NCI Contract 43791 and by funds from the UCSD Cancer Center grant.

REFERENCES

BAKER, R. M., VAN VOORHIS, W. C. & SPENCER, L. A. (1979) HeLa cell variants that differ in sensitivity to monofunctional alkylating agents with independence of cytotoxic and mutagenic responses. *Proc. Natl Adac. Sci. U.S.A.*, **76**, 5249.

BEAVO, J. A., ROGERS, N. L., CROFFORD, O. B., HARDMAN, J. G., SUTHERLAND, E. W. & NEWMAN, E. V. (1970) Effects of xanthine derivatives on lipolysis and on adenosine 3′,5′-monophosphate phosphodiesterase activity. *Molec. Pharmacol.*, **6**, 597.

BOWDEN, G. T., TROSKO, J. E., SHAPAS, B. G. & BOUTWELL, R. K. (1975) Excision of pyrimidine dimers from epidermal DNA and non-semiconservative epidermal DNA synthesis following ultraviolet irradiation of mouse skin. *Cancer Res.*, **35**, 3599.

BOYCE, R. P. & HOWARD-FLANDERS, P. (1964) Release of ultraviolet light-induced thymine dimers from DNA in *E. coli* K-12. *Proc. Natl Acad Sci. U.S.A.*, **51**, 293.

BYFIELD, J. E., CALABRO-JONES, P., MURNANE, J., SEAGREN, S. & WARD, J. F. (1979) Transport-dependent cytotoxicity of water *versus* lipid soluble alkylating agents: Origins of cumulative marrow toxicity. *Proc. Am. Assoc. Cancer Res.*, **20**, 136.

BYFIELD, J. E., LEE, Y. C. & KULHANIAN, F. (1976) X-ray excision repair replication and radiation survival in placental mammal cells. *Int. J. Radiat. Oncol. Biol. Phys.*, **1**, 937.

BYFIELD, J. E., LEE, Y. C. & TU, L. (1977) Molecular interactions between Adriamycin and X-ray damage in mammalian tumor cells. *Int. J. Cancer*, **19**, 186.

CLEAVER, J. E. (1968) Defective repair replication of DNA in *xeroderma pigmentosum*. *Nature*, **218**, 652.

CLEAVER, J. E. (1969) Repair replication of mammalian cell DNA: Effects of compounds that inhibit DNA synthesis or dark repair. *Radiat. Res.*, **37**, 334.

CLEAVER, J. E. (1974) Repair processes for photochemical damage in mammalian cells. *Adv. Radiat. Biol.*, **4**, 1.

COHEN, M. H. (1972) Enhancement of the antitumor effect of 1,3-Bis(2-chloro-ethyl)-1-nitrosourea by vitamin A and caffeine. *J. Natl Cancer Inst.*, **48**, 927.

COHEN, M. H. & CARBONE, P. P. (1972) Enhancement of the anti-tumor effects of 1-3-Bis(2-chloroethyl)-1-nitrosourea and cyclophosphamide by vitamin A. *J. Natl Cancer Inst.*, **48**, 921.

CORNISH, H. H. & CHRISTMAN, A. A. (1957) A study of the metabolism of theobromine, theophylline and caffeine in man. *J. Biol. Chem.*, **228**, 315.

EHMANN, U. K., GEHRING, U. & TOMKINS, G. M. (1976) Caffeine, cyclic AMP and post-replication repair of mammalian cell DNA. *Biochim. Biophys. Acta*, **447**, 133.

FOX, M. & MCMILLAN, S. (1977) Relationship between caffeine sensitive and resistant DNA repair, cell lethality and mutagenesis in mammalian cells after X-rays and alkylating agents. *Stud. Biophys.*, **61**, 71.

FRAVAL, H. N. A. & ROBERTS, J. J. (1979) Excision repair of cis-Diamminedichloroplatinum (II)-induced damage to DNA of Chinese hamster cells. *Cancer Res.*, **39**, 1793.

FUJIMOTO, K., YOSHIDA, S., MORIYAMA, Y. & SAKAGUCHI, T. (1976) Absorption, distribution, excretion and metabolism of 1-(5-oxohexyl) theobromine (BL191) in rats. *Chem. Pharmacol. Bull.*, **24**, 1137.

GAUDIN, D. & YIELDING, L. K. (1969) Response of a "resistant" plasmacytoma to alkylating agents and X-rays in combination with "excision repair" inhibitors caffeine and chloroquine. *Proc. Soc. Exp. Biol. Med.*, **131**, 1413.

GAUTSCHI, J. R., YOUNG, B. R. & PAINTER, R. B. (1972) Evidence for DNA repair replication in unirradiated mammalian cells—Is it an artifact? *Biochim. Biophys. Acta.*, **281**, 324.

GOODMAN, L. S. & GILMAN, A. (1975) *The Pharmacological Basis of Therapeutics*. New York: Macmillan Publ. p. 347.

GOTH, R. & CLEAVER, J. E. (1976) Metabolism of caffeine to nucleic acid precursors in mammalian cells. *Mutat. Res.*, **36**, 105.

KIHLMAN, B. A. (1974) Effects of caffeine on the genetic material. *Mutat. Res.*, **26**, 53.

KIHLMAN, B. A., STURELID, S., HARTLEY-ASP, B. & NILSSON, K. (1974) The enhancement by caffeine of the frequencies of chromosomal aberrations induced in plant and animal cells by chemical and physical agents. *Mutat. Res.*, **26**, 105.

LANG, H. (1975) Model for repair inhibition by caffeine. *Stud. Biophys.*, **50**, 213.

LANG, H. (1976) On the interaction between caffeine and nucleic acids. 1. The influence of caffeine on the secondary structure of native DNA and RNA. *Stud. Biophys.*, **55**, 137.

LEHMANN, A. R. (1974) Minireview—post-replication repair in mammalian cells. *Life Sci.*, **15**, 2005.

MAHER, V. M., OULETTE, L. M., CURREN, R. D. & MCCORMICK, J. J. (1976) Caffeine enhancement of the cytotoxic and mutagenic effect of ultraviolet irradiation in a *xeroderma pigmentosum* variant strain of human cells. *Biochem. Biophys. Res. Commun.*, **71**, 228.

MAHER, V. M., OULETTE, L. M., MITTLESTAT, M. & MCCORMICK, J. J. (1975) Synergistic effect of caffeine on the cytotoxicity of ultraviolet irradiation and of hydrocarbon epoxides in strains of *xeroderma pigmentosum*. *Nature*, **258**, 760.

MARTINDALE (1972) *The Extra Pharmacopoeia*. Ed. Blacow. London: Pharmaceutical Press. p. 350.

MOURELATOS, D. C. (1979) Enhancement by caffeine of sister chromatid exchange frequency induced by anti-neoplastic agents in human lymphocytes. *Experientia*, **35**, 822.

MURNANE, J. P., BYFIELD, J. E., WARD, J. F. & CALABRO-JONES, P. (1981) Effects of methylated xanthines on mammalian cells treated with bifunctional alkylating agents. *Nature*, **285**, 326.

MURNANE, J. P. (1980) The structure of methylated xanthines in relation to their effects on DNA synthesis and cell lethality, alone and in combination with nitrogen mustard. *Biophys. J.*

OGILVIE, R. I. (1978) Clinical pharmacokinetics of theophylline. *Clin. Pharmacokinet.*, **3**, 267.

PERPER, R. J., ZEE, T. W. & MICKELSON, M. W. (1968) Purification of lymphocytes and platelets by gradient centrifugation. *J. Lab. Clin. Med.*, **72**, 842.

RAUTH, A. M. (1967) Evidence for dark-reactivation of ultraviolet light damage in mouse L-cells. *Radiat. Res.*, **31**, 121.

RAUTH, A. M., BARTON, B. & LEE, C. P. Y. (1970) Effects of caffeine on L-cells exposed to mitomycin C. *Cancer Res.*, **30**, 2724.

REGAN, D., TROSKO, J. E. & CARRIER, W. L. (1968) Evidence for excision of ultraviolet-induced pyrimidine dimers from the DNA of human cells *in vitro*. *Biophys. J.*, **8**, 319.

ROBERTS, J. J. (1978) The repair of DNA modified by cytotoxic, mutagenic and carcinogenic chemicals. *Adv. Radiat. Biol.*, **7**, 212.

ROBERTS, J. J., STURROCK, J. E. & WARD, K. N. (1974) The enhancement by caffeine of alkylation-induced cell death, mutations and chromosomal aberrations in Chinese hamster cells as a result of inhibition of post-replication DNA repair. *Mutat. Res.*, **26**, 129.

ROBERTS, J. J. & WARD, K. N. (1973) Inhibition of post-replication repair of alkylated DNA by caffeine in Chinese hamster cells but not HeLa cells. *Chem.-Biol. Interact.*, **7**, 241.

SCHROY, C. B. & TODD, P. (1975) Potentiation by caffeine of ultraviolet damage in cultured human cells. *Mutat. Res.*, **33**, 347.

SETLOW, R. B. & CARRIER, W. L. (1964) The disappearance of thymine dimers from DNA: An error-correcting mechanism. *Proc. Natl Acad. Sci. U.S.A.*, **51**, 226.

SNYDER, M. H., KIMLER, B. F. & LEEPER, D. B. (1977) The effect of caffeine on radiation-induced division delay. *Int. J. Radiat. Biol.*, **32**, 281.

SPRIET, A., SPRIET, C., LAROUSSE, C., CHIGOT, D., ROUX, M. & SIMON, P. (1977) Methodology and results of a survey of adverse reactions to a drug in private practice. *Eur. J. Clin. Pharmacol.*, **11**, 181.

SUGIURA, K., SCHMID, F. T., SCHMID, M. M. & BROWN, G. F. (1972) Effect of compounds on a spectrum of rat tumors. *Cancer Chemother. Rpt* (Part 2), **3**, 231.

TIMSON, J. (1975) Theobromine and theophylline. *Mutat. Res.*, **32**, 169.

TOLMACH, L. J., JONES, R. W. & BUSSE, P. M. (1977) The action of caffeine on X-irradiated HeLa cells. I. Delayed inhibition of DNA synthesis. *Radiat. Res.*, **71**, 653.

TS'O, P. O. P. & LU, P. (1964) Interactions of nucleic acids. I. Physical binding of thymine, adenine steroids, and aromatic hydrocarbons to nucleic acids. *Proc. Natl Acad. Sci. U.S.A.*, **51**, 17.

WALDREN, C. A. & RASKO, I. (1978) Caffeine enhancement of X-ray killing in cultured human and rodent cells. *Radiat. Res.*, **73**, 95.

WALKER, I. G. & REID, B. D. (1971) Caffeine potentiation of the lethal action of alkylating agents on L-cells. *Mutat. Res.*, **12**, 101.

WILKINSON, R., KIEFER, J. & NIAS, A. H. W. (1970) Effects of post-treatment with caffeine on the sensitivity of ultraviolet light irradiation of two lines of HeLa cells. *Mutat. Res.*, **10**, 67.